# Safety of nOPV2 administered during a supplementary immunisation activity in Uganda, 2022: data triangulation from a prospective cohort event monitoring programme and vaccine safety surveillance reports

**DOI:** 10.1016/S2214-109X(25)00110-X

**Published:** 2025-05-22

**Authors:** Ashley T Longley, Fred Nsubuga, Zunera Gilani, Farrell A Tobolowsky, Annet Kisakye, Sharon A Greene, Immaculate Ampeire, Vincent Fred Ssennono, Samuel Ofori Gyasi, Ismail Ntale, Philip Bammeke, Brock Stewart, Helen Byomire Ndagije, Daniel J Kyabayinze, Jane F Gidudu

**Affiliations:** **Centers for Disease Control and Prevention, Atlanta, GA, USA** (A T Longley MPH, Z Gilani PhD, F A Tobolowsky DO, S A Greene PhD, P Bammeke MSc, B Stewart PhD, J F Gidudu MD); **African Field Epidemiology Network, Kampala, Uganda** (F Nsubuga MPH); **Uganda Vaccines and Immunisation Division, Ministry of Health, Kampala, Uganda** (F Nsubuga); **World Health Organization, Kampala, Uganda** (A Kisakye MPH, S O Gyasi MPH); **Uganda National Expanded Program on Immunisation, Uganda Ministry of Health, Kampala, Uganda** (I Ampeire MSc); **Uganda Bureau of Statistics, Kampala, Uganda** (V F Ssennono PhD); **Uganda National Drug Authority, Kampala, Uganda** (I Ntale PharmD, H B Ndagije PhD); **Directorate of Public Health, Uganda Ministry of Health, Kampala, Uganda** (D J Kyabayinze PhD)

## Abstract

**Background:**

In November, 2020, WHO authorised novel oral polio vaccine type 2 (nOPV2) use under Emergency Use Listing in response to outbreaks of circulating vaccine-derived poliovirus type 2 (cVDPV2). Although no concerns were identified in nOPV2 trials, the Global Advisory Committee on Vaccine Safety requested more extensive vaccine safety data during emergency use. The Uganda Ministry of Health declared a cVDPV2 outbreak in 2021 and responded with an nOPV2 campaign in January, 2022. More than 9 million children aged 0–59 months were vaccinated, providing an opportunity to generate robust safety data.

**Methods:**

We monitored the safety of nOPV2 for 42 days post-vaccination using: routine passive surveillance for adverse events following immunisation (AEFI); ongoing acute flaccid paralysis (AFP) surveillance; active, hospital-based surveillance for pre-specified adverse events of special interest (AESI); and active, cohort event monitoring. AFP cases were reviewed by the National Polio Expert Committee. Serious AEFI and all AESI and AFP cases with nOPV2 receipt underwent causality assessment by the National AEFI Committee.

**Findings:**

Across surveillance systems, 1128 children vaccinated with nOPV2 experienced one or more AEFI: 43 children identified through passive surveillance, 128 suspected AFP cases, five AESI cases, and 952 children with reported AEFI through cohort event monitoring. Overall, 109 adverse events were considered serious; six (fever, gastroenteritis (n=3), acute disseminated encephalomyelitis, and encephalitis) were determined by the National AEFI Committee to be consistent with causal association to immunisation with nOPV2. No cases of vaccine-associated paralytic poliomyelitis were detected. One death was detected, considered inconsistent with causal association to immunisation with nOPV2, per the National AEFI Committee.

**Interpretation:**

No new safety concerns were identified with nOPV2 use in Uganda following a national vaccination campaign, providing valuable data that informed WHO prequalification and product licensure.

**Funding:**

Centers for Disease Control and Prevention.

## Introduction

Following the globally synchronised switch from trivalent oral poliovirus vaccine (OPV) to bivalent OPV for use in routine immunisation in 2016, population immunity to type 2 poliovirus declined, and subsequently, outbreaks of circulating vaccine-derived poliovirus type 2 (cVDPV2) increased, starting in 2019.^1,2^ Sabin-strain monovalent type 2 oral poliovirus vaccine (mOPV2) was used in response to cVDPV2 outbreaks, which carried its own risk of seeding new cVDPV2 emergences where population immunity is low.^3^ To accelerate polio eradication, a more genetically stable vaccine, novel oral poliovirus vaccine type 2 (nOPV2), was developed to interrupt cVDPV2 transmission with lower risk of seeding cVDPV2 emergences.^4^

Phase 1–3 clinical trials identified no safety concerns with nOPV2 administration when compared with mOPV2; however, these clinical trials had relatively small sample sizes.^5–8^ In November, 2020, WHO authorised nOPV2 for use in cVDPV2 outbreak response under Emergency Use Listing, during which countries were required to conduct enhanced safety monitoring.^9,10^ Preliminary data from vaccine safety surveillance in the first countries to employ nOPV2 (Liberia, Nigeria, Benin, and Democratic Republic of the Congo) supported findings from clinical trials.^9,11^ However, the Global Advisory Committee on Vaccine Safety requested more comprehensive data to inform WHO prequalification and product licensure and promote vaccine confidence among stakeholders. Specifically, the Global Advisory Committee on Vaccine Safety called for more complete clinical and laboratory data from investigations and causality assessment of serious adverse events following immunisation (AEFI) and adverse events of special interest (AESI), rare and complex adverse events that potentially are causally-associated with poliovirus and poliovirus vaccines.^9,10^ After nearly 1 billion doses administered and enhanced safety monitoring activities during Emergency Use Listing, WHO prequalified nOPV2 in December, 2023.^12^

Comprehensive vaccine safety surveillance data collected in Uganda contributed to the WHO prequalification decision. A polio outbreak was declared in Uganda in July, 2021 following the identification of a cVDPV2 isolate through environmental surveillance in Kampala.^13^ In response, the Uganda Ministry of Health conducted an nOPV2 supplementary immunisation activity (mass immunisation campaign) in January, 2022, vaccinating 9 768 697 children aged 0–59 months with one dose of nOPV2. Alongside the supplementary immunisation activity, we conducted enhanced surveillance nationally to identify and characterise events temporally associated with nOPV2 receipt to generate further data on the vaccine’s safety.

## Methods

### Study design

We assessed the safety of nOPV2 among children aged 0–59 months using the following: routine passive surveillance for AEFI; ongoing passive and active surveillance for acute flaccid paralysis (AFP); active, hospital-based surveillance for pre-specified AESI; and active, cohort event monitoring. Vaccination status was ascertained through child finger-marking or caregiver recall. The surveillance period for all systems was the vaccination date to 42 days post-vaccination. AEFI investigations and causality assessments among vaccinated cases were conducted per WHO guidelines.^14^ The protocol was approved by The Uganda AIDS Support Organization Research Ethics Committee (TASO-2021–70), the Uganda National Council of Science and Technology (HS1972ES), and US Centers for Disease Control and Prevention Human Subjects Office as non-research.

### Passive AEFI surveillance

We reviewed data from the passive (spontaneous) AEFI surveillance system reported during the surveillance period. Per WHO guidelines, AEFI are defined as untoward medical occurrences following immunisation that might or might not have a causal relationship with usage of the vaccine. Serious AEFI are defined as those that result in death, are life-threatening, require inpatient hospitalisation or prolongation of existing hospitalisation, or result in persistent or significant disability or incapacity.^15^

Uganda guidelines require reporting of serious AEFI within 24 h of detection and district AEFI investigation teams initiate case investigations within 48 h of report. AEFI detected at health facilities were reported using the national AEFI reporting form, including the reporter and patient details, facility name, vaccine details (eg, antigen, lot, vaccination date and time), and AEFI signs, symptoms, and clinical outcome. The Uganda National Expanded Program on Immunization analysed reports of serious AEFI for further investigation, per country protocol.^14^ Clinicians, surveillance officers, and caregivers could also report AEFI to the National Drug Authority via a toll-free phoneline, WhatsApp messages, and an SMS-based messaging system.

Provisional diagnoses were determined by treating clinicians. Using WHO guidelines, the National AEFI Committee conducted a causality assessment, a systematic evaluation to determine the likelihood that an event might be causally associated with the vaccine or vaccination.^16^ Causality assessment classified cases into three categories: consistent with causal association to immunisation (ie, vaccine product-related reactions, vaccine quality defect-related reactions, immunisation errors, and anxiety-related reactions); indeterminate (ie, temporally related to vaccination but insufficient evidence to determine causal association with the vaccine); or inconsistent with causal association to immunisation (ie, coincidental).^16^

### AFP surveillance

AFP was defined as sudden onset of weakness or paralysis in any limb.^17^ For this evaluation, we included AFP in a child aged 59 months or younger with symptom onset during January–March, 2022. Surveillance officers or clinicians collected two stool samples 24–48 h apart from all children with AFP and completed the AFP investigation form. The Global Polio Eradication Initiative assesses surveillance quality using a key performance indicator on stool adequacy which is defined as two stool specimens that are collected from patients with AFP within 14 days of paralysis onset, at least 24 h apart, and received in good condition (ie, without leakage or desiccation) by a WHO-accredited laboratory via reverse cold chain (a transportation and storage method designed to keep samples at recommended temperatures from collection through to arrival at the laboratory). A 60-day follow-up is conducted for all AFP cases with inadequate stool specimens because the presence of residual paralysis could be due to poliovirus. We expanded this practice to include all AFP cases with nOPV2 detected in stool specimens to determine if paralysis was present. The stool samples were transported to the Uganda Virus Research Institute in reverse cold chain (2–8°C) to test for poliovirus and other non-polio enteroviruses. Positive poliovirus isolates were sent to the National Institute for Communicable Diseases in South Africa for sequencing.

AEFI investigation teams conducted detailed case investigations for each suspected AFP case with nOPV2 receipt to identify provisional diagnoses. The National Polio Expert Committee (NPEC) reviewed all AFP investigation forms and corresponding virological data. All suspected AFP case-patients with nOPV2 receipt with pre-existing medical conditions (not acute onset) that could cause weakness were excluded from analysis because they did not meet the case definition of a true AFP case. The causality committee reviewed all remaining AFP cases with nOPV2 receipt with residual paralysis for evidence of vaccine-associated paralytic polio. Vaccine-associated paralytic polio was defined as a case of AFP with residual paralysis (compatible with paralytic poliomyelitis) lasting at least 60 days and occurring in an nOPV2 recipient between 4 days and 40 days after the dose of nOPV2 was administered. The National Polio Expert Committee reviewed and classified all AFP cases as polio-compatible based on available clinical information or discarded as non-polio AFP.

### AESI surveillance

We conducted prospective, active hospital-based surveillance for AESI in 14 regional referral hospitals and four tertiary hospitals in Kampala, all high-volume facilities with capacity to diagnose complex conditions. We included anaphylaxis, aseptic meningitis, encephalitis, acute disseminated encephalomyelitis, Guillain-Barré syndrome and Fisher Syndrome, myelitis and transverse myelitis, AFP (including vaccine-associated paralytic polio), and unexplained death.^18^

Integrated Disease Surveillance and Response Officers visited each facility weekly to review registers from all inpatient and outpatient departments that manage children aged 59 months and younger. Using paper-based symptom screening tools and line listing forms, Integrated Disease Surveillance and Response Officers identified suspected AESI cases among admitted and recently discharged patients, regardless of vaccination status, then ascertained nOPV2 status by checking finger-marking or by calling the caregivers for verbal recall.

Facility paediatricians abstracted medical information from nOPV2-vaccinated patient charts using standardised abstraction forms, collecting patient demographics, medical history, and relevant clinical findings (eg, physical examinations, laboratory, and radiographic diagnostic testing). The Global Polio Eradication Initiative AESI surveillance guide and standardised Brighton Collaboration case definitions, when applicable, were used to confirm diagnoses and assign levels of diagnostic certainty, a measure of the specificity of the AESI.^18^

Case-patients diagnosed with a pre-specified AESI who received nOPV2 during the supplementary immunisation activity were treated as serious AEFI, undergoing investigation and causality assessment, as described above.

### Cohort event monitoring

We conducted cohort event monitoring surveillance on a nationally representative cohort of nOPV2 recipients for 42 days following the date of vaccination. Using a two-stage cluster sampling method to identify our population, we estimated the sample size assuming a conservative estimated nOPV2 coverage of 50%, a one-sided CI of 5%, and 30% non-response rate.^19^ The resulting sample size was 2000 households. Sampling was conducted by the Uganda Bureau of Statistics. The size of the primary sampling unit, enumeration areas, was estimated using projections from 2014 census data. We sampled 200 of 78 692 enumeration areas using probability proportional to estimated size. Before the campaign, enrolment officers listed households in the selected enumeration areas. Uganda Bureau of Statistics then systematically selected ten households from each enumeration area without replacement.

Enrolment officers visited the selected households following the vaccination teams to enrol eligible children who planned to reside in the selected communities for at least 42 days after nOPV2 vaccination, had no acute signs or symptoms at the time of vaccination, and had a caregiver with access to a phone who planned to stay with the child for at least 42 days after vaccination and provided written informed consent. During enrolment (day 0), demographics, health status, underlying medical conditions, and vaccination history were collected from caregivers. Telephone health check-ins occurred on days 3, 7, 14, 28, and 42 post-vaccination. Using a standardised questionnaire, data collectors solicited a list of adverse events (including free responses) since last contact and type of health care sought, when applicable. Phone call attempts were made three times over a two-day period before the child was considered lost to follow-up for that check-in point. Data collectors resumed calls at the subsequent check-in point. Data collectors entered data on paper and into Open Data Kit software.

### Data analyses

Routine data harmonisation meetings were held to compare and reconcile reported AEFI data across surveillance systems. Participant characteristics, AEFI (serious and non-serious), and causality assessment classifications were summarised using descriptive statistics. Rates of AESI were calculated per 100 000 doses of nOPV2 administered. Analyses were conducted in Excel and SAS 9.4.

### Role of the funding the source

The funder of the study was involved in programme design, data collection, data analysis, data interpretation, and writing of the report.

## Results

Overall, 1128 children vaccinated with nOPV2 experienced one or more AEFI across the four surveillance systems: 43 children were identified through passive surveillance, with two (5%) of 43 cases characterised as serious; 128 suspected AFP cases were identified through the AFP surveillance system; five AESI cases were identified through the hospital-based surveillance (two of which were also identified via AFP surveillance); and 952 children had AEFI reported through cohort event monitoring, with 22 (2%) cases characterised as serious (figure).

Among the 9 768 697 nOPV2 vaccine doses administered, 43 children experiencing one or more AEFI were reported through passive AEFI surveillance (70 total AEFI reported); 29 (67%) of 43 were male and the median age was 36 months (IQR 24–48; table 1). 20 (47%) of 43 children reportedly experienced more than one AEFI, and more than half of children reported fever (n=25; 58%). Other common reports included cough (n=17; 40%), coryza (n=10; 23%), and diarrhoea (n=8; 19%). Two serious AEFI were identified, meningitis and leg weakness, with onset at 11 days and 12 days following nOPV2 vaccination, respectively. The meningitis case was classified as inconsistent causal association to immunisation (coincidental) per the National AEFI Committee (table 2). The 24-month-old child with leg weakness was lost to follow-up before an investigation could be made; this case was not detected through other surveillance systems.

Overall, 159 suspected AFP cases were reported through national AFP surveillance. Two of these cases were also identified via hospital-based surveillance with different diagnoses. Caregivers reported that 128 (81%) of 159 received nOPV2 in the supplementary immunisation activity; 81 (63%) were male and the median age was 30 months (IQR 18–41; table 1). Virological data showed that 17 (11%) AFP cases had nOPV2, two (1%) had non-polio enterovirus, and one (<1%) had type 3 Sabin OPV isolated from stool.

All AFP cases with nOPV2 isolated from specimens were considered adequate, six had follow-up investigation, and two had residual paralysis that were classified as discarded (non-polio) by the NPEC because the detailed investigation attributed the underlying cause to be injection neuritis. 22 (14%) of 159 AFP cases had inadequate stool specimens and 16 (73%) of 22 had completed 60-day follow-up examinations. Five of these cases had residual paralysis but were all classified as discarded based on clinical data review.

The 128 AFP cases with nOPV2 receipt had detailed AEFI investigations: 47 cases were removed from analysis because the onset of the reported medical event occurred before vaccination. 78 (96%) of the remaining 81 cases were classified as coincidental events, and one was unclassifiable due to insufficient information (table 2). The most common coincidental findings included transient limb paralysis (8 [10%] of 81) and injection neuritis (23 [28%] of 81), determined to be caused by injectable diclofenac administered to treat fever following vaccination. Of 128 AFP cases that underwent causality assessment, two events (2%) were classified by the National AEFI Committee as consistent with causal association to immunisation and vaccine product-related reactions, with the final diagnoses being fever and acute gastroenteritis. Both cases yielded negative poliovirus test results and were discarded as non-polio AFP cases by the NPEC. No AFP cases were characterised as vaccine-associated paralytic polio.

Sensitivity analysis of the 33 AFP cases with no or unknown nOPV2 status showed similar demographic and clinical characteristics to cases with nOPV2 receipt. However, more children who received nOPV2 had documented OPV in routine immunisation (n=17, 14%) compared with children without confirmed nOPV2 receipt (n=8, 24%; appendix 2 p 1).

Eight children with one AESI each were detected through hospital-based surveillance. Three children with aseptic meningitis were unvaccinated. Among the five (63%) children vaccinated with nOPV2, four (80%) of five were male, and the median age was 6 months (IQR 2–48 months; table 1). The AESI included aseptic meningitis (n=1), acute disseminated encephalomyelitis (n=1), and encephalitis (n=3), with a median symptom onset of 12 days following vaccination (range 3–41 days; table 3). Three (60%) cases were classified as inconsistent with causal association to immunisation (coincidental). The remaining two (40%) cases, acute disseminated encephalomyelitis and encephalitis, were classified as consistent with vaccine product-related reactions (table 2). The crude reporting rates of aseptic meningitis, acute disseminated encephalomyelitis, and encephalitis among the vaccinated population (regardless of causality classification) were 0·01, 0·3, and 0·1 per 100 000 nOPV2 supplementary immunisation activity doses administered, respectively. Among those determined to be causally consistent, the rates of acute disseminated encephalomyelitis and encephalitis were both 0·1 per 100 000 doses administered.

Cohort event monitoring enumerators visited 1889 households; approximately 5% of enumeration areas had inaccurate geolocation or were not accessible. A caregiver from 1796 (95%) of 1889 households consented to participate and 1592 (89%) of 1796 households had eligible children. Overall, 2213 (98%) of 2258 eligible and enrolled children were reachable for follow-up and included in the AEFI analysis; 1114 (50%) of 2213 were males and the median age was 31 months (IQR 17–44). Common reasons for ineligibility among households included lack of age-eligible children (154 [31%] of 495), presence of acute signs and symptoms at vaccination (146 [29%] of 495), and lack of phone access (73 [15%] of 495; appendix 2 p 3).

Among the 2213 children followed up, caregivers of 952 participants (43%) reported one or more AEFI during the surveillance period (1376 total AEFI reported); 486 (51%) children with AEFI reports were male, and the median age was 30 months (IQR 16–42; table 1). The most reported AEFI were fever (n=463/2213, 21%), diarrhoea (n=171/2213, 8%), and general malaise (n=131/2213, 6%). Most AEFI (n=713/1376, 52%) were reported within the first week of vaccination (table 4).

Among the AEFI reports through cohort event monitoring, 22 were classified as serious, all from different enumeration areas. The median age of case-patients was 26 months (IQR 12–43) and more than half were male (n=13; 59%). The most frequent final diagnoses for the case-patients with serious AEFI included malaria (n=5; 23%), acute febrile illness requiring hospitalisation (n=3; 14%), and pneumonia (n=3; 14%). 21 children had an overnight stay at a hospital or health facility; most (n=15; 68%) were hospitalised within 7 days of vaccination (appendix 2 p 2). One case-patient died, diagnosed with severe oedematous malnutrition. 20 of these cases were determined to be inconsistent with causal association to immunisation and two were classified as vaccine product-related reactions, both with diagnoses of gastroenteritis (table 2).

## Discussion

No unexpected signals were identified through triangulation of data from four vaccine safety surveillance systems. We leveraged existing surveillance systems and implemented additional active surveillance to generate a more comprehensive understanding of nOPV2 safety. With the addition of cohort event monitoring, a method beyond the Emergency Use Listing monitoring requirements, we actively followed up a sample of children to more completely document non-serious and serious AEFI than passive surveillance. The vast majority of reported AEFI across surveillance systems were coincidental, and no cases of AFP were attributed to vaccine-associated paralytic polio.

The safety profile from this evaluation is similar to clinical trial and initial use data, demonstrating an absence of substantive safety concerns.^5,6,8^ The majority of AEFI identified were coincidental and non-serious, consistent with data submitted from 13 countries that have used the vaccine.^5–7^ The findings indicate that non-serious AEFI are common—mostly reports of fever and gastrointestinal issues that also could have been coincidental. This is consistent with the literature on AEFI reported with Sabin strain OPVs.^8,20^ Most reported fevers occurred within 3 days of vaccination, which is common following immunisations.^21^ Notably, especially at later timepoints, fevers among children aged 0–59 months in Uganda could be due to other causes (eg, malaria and respiratory illnesses).

Importantly, no cases of vaccine-associated paralytic polio were detected, as determined by the NPEC and the National AEFI Committee, a level of review not conducted in routine surveillance. Per the NPEC, there was sufficient evidence from the case investigations to conclude that the observed residual paralysis was not consistent with poliomyelitis or vaccine-associated paralytic polio due to nOPV2 receipt. Although vaccine-associated paralytic polio can rarely occur following administration of Sabin strain OPVs, operational data suggest that nOPV2 has lower rates of vaccine-associated paralytic polio compared with previous Sabin strain OPVs.^22^ Our findings are consistent with those seen in both clinical trials and from initial use data.^5,23^

Understanding the expected, non-serious AEFI can help address vaccine hesitancy and non-compliance. Simultaneously, serious AEFI or AESI, even if coincidentally associated, can jeopardise trust in the immunisation programne. Most safety events reported from the four surveillance systems were coincidental with nOPV2 receipt, similar to the majority of published studies that report no causally associated serious AEFI with nOPV2.^23^ Among the serious AEFI detected, only six cases were classified as vaccine product-related reactions. The cases of gastroenteritis determined to be causally associated were not unexpected as diarrhoea, vomiting, and abdominal pain have been reported with OPV receipt from several studies.^5,8,20^

Under Emergency Use Listing, countries deploying nOPV2 were required to monitor for pre-specified AESI, as there is evidence that live vaccines, including OPVs, can very rarely lead to neurological AEFI.^24,25^ Cases of aseptic meningitis were detected among unvaccinated children, though, highlighting the importance of background data for interpretation of vaccine safety results. Rates of the pre-specified AESI are unavailable from low-resource settings, making it difficult to evaluate potential excess cases of AESI versus the expected rates in the absence of nOPV2. Of note, the available medical information from our hospital-based surveillance was insufficient to confirm the diagnoses according to the Brighton Collaboration criteria; therefore, we relied upon physician diagnoses. Nevertheless, given the frequency of serious events occurring in the population regardless of vaccination, the importance of background rates and detailed AEFI investigations cannot be understated.

The Global Advisory Committee on Vaccine Safety recommends that AFP surveillance serve as the backbone of nOPV2 safety surveillance, a sensitive surveillance system that captures conditions of interest that are, or could resemble, paralytic polio (eg, vaccine-associated paralytic polio or Guillain-Barré syndrome).^9^ This infrastructure further proved beneficial in identifying a cluster of injection neuritis cases due to improper injection. Leveraging the AFP surveillance platform for surveillance of other vaccine-preventable diseases and AEFI and AESI could improve vaccine safety monitoring generally.^26^ Now that nOPV2 has been prequalified by WHO, sensitive AFP surveillance will continue to serve as the cornerstone activity to detect presence or absence of circulating poliovirus as well as safety signals following administration.

Our evaluation was subject to limitations. Under-reporting of AEFI is a general limitation of passive surveillance. AEFI identified from the cohort event monitoring were caregiver-reported without clinical validation, so there was potential for over-reporting, although most of these AEFI were non-serious. Similarly, AFP cases are often reported more frequently during a supplementary immunisation activity when teams actively search for AFP cases, leading to possible over-reporting and difficulty in distinguishing true AFP cases from non-acute and non-flaccid paralysis. However, among reported AFP cases, many had underlying conditions or were not temporally associated with vaccination. For suspected AFP cases that met the case definition, most had a detailed follow-up investigation and enough evidence to assess for vaccine-associated paralytic polio, although we did not assess for contact vaccine-associated paralytic polio, which can occur among close contacts of an OPV recipient between 7 days and 60–75 days after OPV administration.^22^ Several case investigations for serious AEFI detected in remote districts were delayed, which could have resulted in a loss of some available clinical data. Lastly, with finger-marking the only form of validation of recent vaccination, there was potential for some verbally reported vaccinations to be inaccurate.

The supplementary immunisation activity in Uganda, vaccinating over 9 million children, provided a unique opportunity to conduct a comprehensive safety evaluation, adding to the growing evidence of nOPV2 safety and providing immunisation stakeholders globally with important information for decision making. These data, including the lack of unexpected safety signals, are important to help advance polio eradication goals of reducing paralytic polio and ultimately replacing live attenuated OPVs with inactivated poliovirus vaccine. Additional efforts to strengthen vaccine safety surveillance throughout the African region, including estimating background rates of AESI, validating the Brighton Collaboration case definitions in low-resource settings, and leveraging AFP surveillance platforms, will further strengthen the safety data on nOPV2 use.

## Supplementary Material

SM1

SM2

SM3

## Figures and Tables

**Figure:**
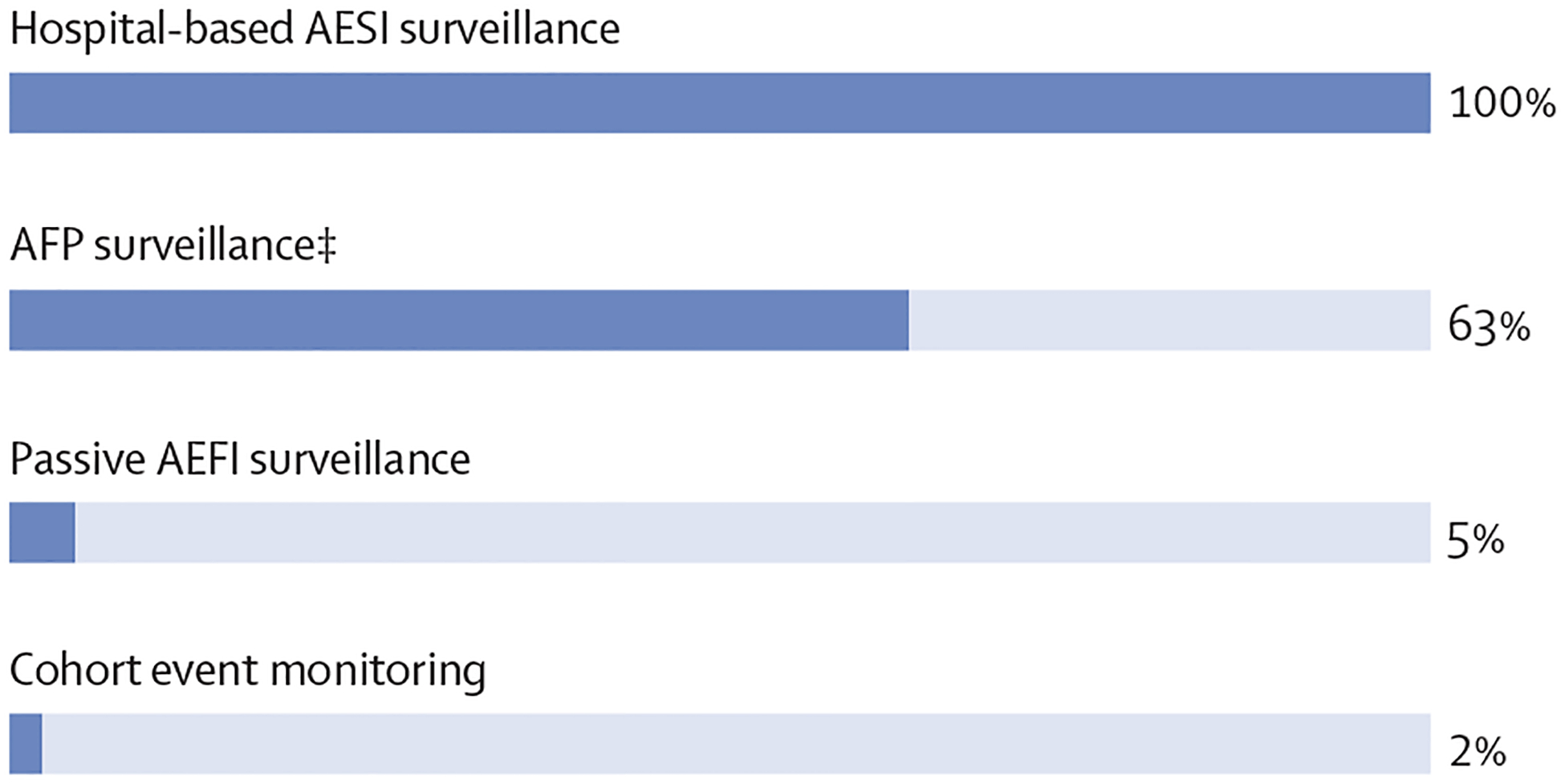
Percentage of children* with reported serious AEFI or AFP, by surveillance type†, following the nOPV2 supplementary immunisation activity—Uganda, January, 2022 AEFI=adverse events following immunisation. AESI=adverse events of special interest. AFP=acute flaccid paralysis. nOPV2=novel oral poliovirus vaccine type 2. *Child could have multiple reported AEFI. †Two children identified through hospital-based surveillance for AESI were also reported through routine AFP surveillance. The cases are included in each surveillance stream in this figure. All cases of AFP that underwent causality assessment by the National AEFI Committee were considered serious events. ‡47 suspected AFP cases were detected that upon further investigation did not meet the AFP case definition of sudden onset or flaccid and were categorised as non-serious.

**Table 1:** Demographic characteristics of children* vaccinated with nOPV2 detected through the AEFI or AFP surveillance systems, by surveillance type, nOPV2 supplemental immunisation activity—Uganda, January, 2022

	Passive AEFI surveillance	Hospital-based AESI surveillance	AFP surveillance	Cohort event monitoring
Number of children reporting AEFI†	43	5	128	952
Sex				
Male	29 (67%)	4 (80%)	81 (63%)	486 (51%)
Female	14 (33%)	1 (20%)	47 (37%)	466 (49%)
Median age, months	36 (24–48)	6 (2–48)	30 (18–41)	30 (16–42)
Age group, months				
0–11	3 (7%)	3 (60%)	18 (14%)	155 (16%)
12–23	6 (14%)	0	22 (17%)	231 (24%)
24–59	34 (79%)	2 (40%)	88 (69%)	566 (60%)

Data are n, n (%), or median (IQR). AEFI=adverse events following immunisation. AESI=adverse events of special interest. AFP=acute flaccid paralysis. nOPV2=novel oral poliovirus vaccine type 2.

* Child could be reported through more than one surveillance system.

† One AEFI report per child; report can contain one or more AEFI. Reports of raw data, before any investigations.

**Table 2:** Causality assessment categorisation* among nOPV2-vaccinated cases of serious AEFI and AFP, nOPV2 supplemental immunisation activity— Uganda, January, 2022

	Total	Passive AEFI surveillance†	Hospital-based AESI surveillance	AFP surveillance	Cohort event monitoring
AEFI cases reviewed by causality committee	157	2	5	128	22
Consistent with causal association to immunisation					
Vaccine product-related reaction	6	0	2	2	2
Vaccine quality defect-related reaction	0	0	0	0	0
Immunisation error-related reaction	0	0	0	0	0
Immunisation anxiety-related reaction	0	0	0	0	0
Indeterminate					
Temporal relationship is consistent but there is insufficient definitive evidence for vaccine event	0	0	0	0	0
Qualifying factors result in conflicting trends of consistency and inconsistency with causal association to immunisation	0	0	0	0	0
Inconsistent with causal association to immunisation‡	102	1	3	78	20
Unclassifiable†	2	1	0	1	0
Excluded (not temporally associated with vaccination)	47	0	0	47¶	0

AEFI=adverse events following immunisation. AESI=adverse events of special interest. AFP=acute flaccid paralysis. nOPV2=novel oral poliovirus vaccine type 2.

* Using WHO Causality Assessment guidelines.

† Coincidental underlying or emerging condition(s), or condition(s) caused by exposure to something other than the vaccine.

‡ One serious AEFI was lost to follow-up before investigation and therefore was not reviewed by the causality assessment committee.

¶ Serious adverse events that were excluded from analysis did not go for causality assessment.

**Table 3:** AESI identified among children vaccinated with nOPV2 from prospective hospital-based surveillance, nOPV2 supplemental immunisation activity—Uganda, January, 2022 (n=5)

	Final diagnosis*	Symptom onset from nOPV2 vaccination (days)	Brighton Collaboration levels of diagnostic certainty†	Causality assessment classification
Aseptic meningitis	Aseptic meningitis	6	Category 3: insufficient evidence‡	Coincidental
ADEM	ADEM	3	Category 4: insufficient evidence	Vaccine product-related reaction
Encephalitis	Encephalitis	12	Category 4: insufficient evidence	Coincidental
Encephalitis	Cerebral sinus thrombosis	16	Category 4: insufficient evidence	Coincidental
Encephalitis	Encephalitis	41	Category 2: probable case	Vaccine product-related reaction

ADEM=acute disseminated encephalomyelitis. AESI=adverse events of special interest. nOPV2=novel oral poliovirus vaccine type 2.

* Final diagnoses available following causality assessment.

† Brighton Collaboration levels of diagnostic certainty are a measure of the specificity of the case definition, with level 1 being the highest specificity, a confirmed case, level 2 being a probable case, and level 3 (where applicable) being the least specific (a possible case, most sensitive).

‡ Aseptic meningitis only has two levels, 1 and 2. Level 3 for this condition is a clinician-diagnosed case with insufficient evidence to meet the case definition.

**Table 4:** Number of caregiver-reported AEFI* at each timepoint following vaccination in cohort event monitoring programme†, nOPV2 supplemental immunisation activity—Uganda, January, 2022

	Overall (N=2213)	Day 3 (n=2141)	Day 7 (n=2178)	Day 14 (n=2176)	Day 28 (n=2136)	Day 42 (n=2108)
Any symptoms	1376 (62%)	389 (18%)	411 (19%)	211 (10%)	333 (16%)	174 (8%)
Systemic reactions						
Fever	463 (21%)	140 (7%)	97 (4%)	79 (4%)	89 (4%)	58 (3%)
Headache	61 (3%)	17 (1%)	14 (1%)	16 (1%)	11 (1%)	3 (<1%)
Fatigue	40 (2%)	7 (<1%)	6 (<1%)	10 (<1%)	5 (<1%)	12 (1%)
Irritability	34 (2%)	6 (<1%)	4 (<1%)	13 (1%)	3 (<1%)	8 (<1%)
Chills	27 (1%)	6 (<1%)	4 (<1%)	5 (<1%)	8 (<1%)	4 (<1%)
Persistent crying	24 (1%)	5 (<1%)	1 (<1%)	7 (<1%)	5 (<1%)	6 (<1%)
Myalgia	3 (<1%)	1 (<1%)	0	1 (<1%)	1 (<1%)	0
Gastrointestinal reactions						
Diarrhoea	171 (8%)	53 (2%)	35 (2%)	33 (2%)	30 (1%)	20 (1%)
Nausea or vomiting	79 (4%)	28 (1%)	22 (1%)	10 (<1%)	10 (<1%)	9 (<1%)
Abdominal pain	43 (2%)	10 (<1%)	10 (<1%)	8 (<1%)	7 (<1%)	8 (<1%)
Other reactions						
General malaise	131 (6%)	42 (2%)	31 (1%)	17 (1%)	24 (1%)	17 (1%)
Neck stiffness	1 (<1%)	0	0	1 (<1%)	0	0
Weakness of arms	0	0	0	0	0	0
Weakness of legs	4 (<1%)	3 (<1%)	0	0	1 (<1%)	0
Other‡	295 (13%)	67 (3%)	104 (5%)	21 (1%)	81 (4%)	22 (1%)

Data are n (%). AEFI=adverse event following immunisation. nOPV2=novel oral poliovirus vaccine type 2.

* Child could have experienced one or more AEFI at any timepoint.

† Total vaccinated children followed up=2213; 1376 reported AEFI among 952 children.

‡ Others included any caregiver-reported illness. Common reports included cough, influenza-like symptoms, malaria, and rash.

## Data Availability

The de-identified individual participant data collected for the cohort event monitoring programme and reported in this Article are stored in a non-publicly available repository at Centers for Disease Control and Prevention. AEFI surveillance data and the hospital-based AESI datasets belong to the Ministry of Health of Uganda. Researchers who would like access to these datasets can submit a request to the corresponding author detailing the intended use for the data including primary research questions. All requests must be approved by the Ministry of Health of Uganda. Researchers given access to the data will sign data sharing agreements which will restrict the use to answering pre-specified research questions. The AFP surveillance data are available in the public domain.

## Research in context

### Evidence before this study

Outbreaks of circulating vaccine-derived poliovirus type 2, occurring in settings with low poliovirus immunity, threaten the global polio eradication efforts. In 2022, WHO authorised use of a novel type 2 oral polio vaccine (nOPV2) under Emergency Use Listing to combat these outbreaks. Robust data on the safety and effectiveness of nOPV2 were required to inform WHO prequalification and product licensure for introduction worldwide. We conducted a review of the literature to identify nOPV2 safety data generated during the Emergency Use Listing period. We searched PubMed from 2017 to 2022 using the following terms: “nOPV2”, “novel type 2 oral poliovirus vaccine”, “vaccin* safety”, “safety”, “adverse events following immunization”, “AEFI”, “immunization safety”, “tolerability”, “adverse events of special interest”, and “AESI”, with no language restrictions. We identified ten texts that were primarily findings from clinical trials. Two were studies on genetic characterisation and neurovirulence. No studies were found reporting the safety of nOPV2 outside of a clinical trial setting during the Emergency Use Listing period.

### Added value of this study

This multipronged surveillance generated robust nOPV2 vaccine safety data from both active and passive safety surveillance among millions of children aged 0–59 months in Uganda in a mass vaccination campaign, including case-specific causality assessments. The findings from multiple surveillance systems provide further evidence on the safety profile of nOPV2. Furthermore, we showed the benefit of leveraging existing acute flaccid paralysis surveillance as a platform for vaccine safety monitoring.

### Implications of all the available evidence

The nOPV2 safety data generated in our setting contribute to the growing body of evidence on the vaccine’s safety and validate the absence of new vaccine safety signals to date. These findings informed WHO’s decision to license nOPV2 use under the WHO prequalification framework, and will support country-specific vaccine introduction decisions, with the ultimate goal of supporting global poliovirus eradication.
