# Supplementary material for: Safety of nOPV2 administered during a supplementary immunisation activity in Uganda, 2022: data triangulation from a prospective cohort event monitoring programme and vaccine safety surveillance reports
Source: Lancet Glob Health. Author manuscript; Available in PMC 2025 Jul 2. (PMC12213129; doi:10.1016/S2214-109X(25)00110-X)
Supplement: SM1 [file NIHMS2090134-supplement-SM1.pdf]

THE LANCET
Global Health

Supplementary appendix 3

This Equitable Partnership Declaration (EPD) was submitted by the authors, and we reproduce it as supplied. It has not been peer reviewed. *The Lancet*'s editorial processes have not been applied to the EPD.

Supplement to: Longley AT, Nsubuga F, Gilani Z, et al. Safety of nOPV2 administered during a supplementary immunisation activity in Uganda, 2022: data triangulation from a prospective cohort event monitoring programme and vaccine safety surveillance reports. *Lancet Glob Health* 2025; published online May 22. https://doi.org/10.1016/S2214-109X(25)00110-X.

Equitable Partnership Declaration questions

This Equitable Partnership Declaration is a statement being published online alongside papers at *The Lancet Global Health*, as a separate appendix, to allow researchers to describe how their work engages with researchers, communities, and environments in the countries of study. This is part of our broader goal to decolonise global health, handing control and leadership of research to academics and clinicians who are based in the regions of study, and to affected communities.

Please answer all questions with as much detail as possible, noting that all included information will be published open-access and it will be freely available online to all who wish to read it. If a question does not apply to your study, please state "Not applicable".

The format of and questions in this statement are currently in a pilot phase. Please email Dr Liam Messin (Liam.Messin@lancet.com; deputy editor) and Dr Kate McIntosh (Kate.McIntosh@lancet.com; senior editor) with any feedback, particularly if you find any questions unclear.

Researcher considerations

1. Please detail the involvement that researchers who are based in the region(s) of study had during a) study design; b) clinical study processes, such as processing blood samples, prescribing medication, or patient recruitment; c) data interpretation; and d) manuscript preparation, commenting on all aspects. If they were not involved in any of these aspects, please explain why.

This question is intended for international partnerships; if all your authors are based in the area of study, this question is not applicable.

This should include a thorough description of their leadership role(s) in the study. Are local researchers named in the author list or the acknowledgements, or are they not mentioned at all (and, if not, why)? Please also describe the involvement of early career researchers based in the location of the study. Some of this information might be repeated from the Contributors section in the manuscript. Note: we adhere to ICMJE authorship criteria when deciding who should be named on a paper.

a) Study design:
The study team included epidemiologists, immunization officers, vaccine safety experts and biostatisticians based in Atlanta and Uganda, the setting of the study.

The study was conceptualized because there was CDC funding available to support a comprehensive safety surveillance study at the time the novel oral poliovirus vaccine was approved under EUL. CDC had a long-standing collaboration and ongoing safety surveillance projects with the Ministry of Health and the EPI program in Uganda. When the type 2 poliovirus was detected, this was an opportunity to expand on the existing safety surveillance portfolio.

ATL, FN, SAG, HBN, DJK, JFG conceptualized this study. ATL, FN, ZG, FAT, SAG, VFS, and BS contributed to the final methodology and study design.

FAR, HBN, DJK, and VFS are based in Uganda.

**b) Clinical study processes:**<br>The team of co-investigators led by FN, ZG, FT, PB, AK, and DJK carried out the project implementation activities, project administration, and supervision. Recruitment and training of Cohort Event Monitoring enumerators was done by FN, ZG, and VFS. PB developed the Open Data Kit (ODK) application and carried out the training with enumerators; PB also implemented daily data validation checks. BS and VFS developed the sampling frame. FN, JFG, ZG, and FT provided oversight and supervision of the implementation of each of the study components.<br><br>FN, AK, VFS, and DJK are based in Uganda.
**c) Data interpretation:**<br>Data collection and management was led by FN, ZG and PB, assisted by ATL, ZG, and SAG. Data validation and harmonization was done by FN, IA, VFS, PB, SOG, HBN.<br><br>FN, IA, VFS, SOG, and HBN are based in Uganda.
**d) Manuscript preparation:** The manuscript was developed by FN and ATL with input by ZG, FAT, AK, SAG, IA, VFS, SOG, IN, PB, HBN. ATL, SAG, DJK, JFG edited and reviewed final drafts.<br><br>FN, AK, IA, VFS, SOV, IN, DJK, and HBN are based in Uganda.

2. Were the data used in your study collected by authors named on the paper, or have they been extracted from a source such as a national survey? ie, is this a secondary analysis of data that were not collected by the authors of this paper. If the authors of this paper were not involved in data collection, how were data interpreted with sufficient contextual knowledge?

   The Lancet Global Health *believe contextual understanding is crucial for informed data analysis and interpretation.*

The data used for analysis in this paper includes both primary and secondary data collection:<br>Primary data collection includes:<br>(1) Data collected from the cohort event monitoring study,<br>(2) AEFI case investigation for all AFP cases<br>(3) Causality assessment of all AFP cases, AESI, and serious AEFI cases<br><br>Secondary analysis of data includes:<br>(4) Surveillance data from AFP, passive AEFI, and sentinel hospital-based AESI systems<br>(5) AFP case investigation and stool culture and sequencing results<br>(6) AEFI/AESI case investigation and serious AEFI cases (not including AFP cases)<br><br>Authors on this paper include experts and practitioners from the Uganda Ministry of Health including from the Vaccines and Immunization Division, Bureau of Statistics, Expanded Program on Immunization, and National Drug Authority as well as the African Field Epidemiology Network (AFENET) who aided in data collection and interpretation with awareness of and lived experience of the contextual factors that are critical to interpretation of the data.

3. How was funding used to remunerate and enhance the skills of researchers and institutions based in the area(s) of study? And how was funding used to improve research infrastructure in the area of study?

   Potentially effective investments into long-term skills and opportunities within institutions could include training or mentorship in analytical techniques and manuscript writing, opportunities to lead all or specific aspects of the study, financial remuneration rather than requiring volunteers, and other professional development and educational opportunities.

   Improvements to research infrastructure could be funding of extended trial designs (such as platform trials) and use of master protocols to enable these designs, establishment of long-term contracts for research staff, building research facilities, and local control of funding allocation.

**Skills:**<br>This project began as a collaboration between the Vaccine Safety Team within the Immunization Services Branch of the Global Immunization Division (GID) at CDC led by JG and supported by ZG and ATL with the Polio Eradication Branch in GID and supported by SG and FAT. There were skill and knowledge transfers across the CDC teams. JG had a long-standing working relationship with FN, AK, IA, VFS, SOG, IN, HBN, and DJK given prior vaccine safety study on fractional yellow fever trial in Uganda and Hospital-Based Sentinel Surveillance Study for Adverse Events of Special Interest (HBSS-AESI) which supports vaccine introduction. ATL, FN, SAG, ZG, FAT, PB, and SOG were junior researchers and were supported by JG, DJK, AT, and IA to strengthen fieldwork and study implementation skills.
**Research infrastructure:**<br>This work helped to carry forward the efforts done by the Uganda Vaccines and Immunization Division within the Ministry of Health (MOH) as well as strengthen the safety surveillance capacity led by the Uganda National Drug Authority (NDA). As new vaccines are introduced and target populations throughout the life course, there is increasing need to fortify vaccine safety surveillance infrastructure, particularly in resource constrained settings. This study highlighted how passive AEFI surveillance can be bolstered by sentinel surveillance and CEM. Further, this type of collaboration reinforces relationship between the NDA, MOH, and the expanded program on immunization.

4. How did you safeguard the researchers who implemented the study?

   Please describe how you guaranteed safe working conditions for study staff, including provision of appropriate personal protective equipment, protection from violence, and prevention of overworking.

The study was conducted in accordance with established institutional policies and procedures that safeguard the safety and well-being of study staff and participants. All participating institutions have policies on safe-working conditions and equal employment opportunities that were adhered to during the course of this study.

Benefits to the communities and regions of study

5. How does the study address the research and policy priorities of its location?

How were the local priorities determined and then used to inform the research question? Who decided which priorities to take forward? Which elements of the study address those priorities?

> Many low and middle-income countries lack the resources required to support robust or mature vaccine safety surveillance and vaccine pharmacovigilance program. This challenge is underscored during a response to a public health emergency of international concern. In an effort to address these gaps and generate much needed data for national-level and international-level decision-making, Ministry of Health, the EPI program, and CDC partnered to support the implementation of a new vaccine during an outbreak response and to support overall surveillance activities for AEFI and AESI in Uganda. The results of the safety study in Uganda were presented by the Uganda Ministry of Health and US Centers for Disease Control and Prevention to the Global Advisory Committee on Vaccine Safety Sub-Committee on novel type 2 oral poliovirus vaccine (nOPV2) Safety in January 2023.

6. How will research products be shared in the community of study?

For instance, will you be providing written or oral layperson summaries for non-academic information sharing? Will study data be made available to institutions in the region(s) of study? The Lancet Global Health *encourages authors to translate the summary (abstract) into relevant languages after paper editing; do you intend to translate your summary?*

> The team is committed to long-term immunization systems strengthening work including vaccine safety and supporting national regulatory authorities to facilitate national policy making on vaccine introduction and vaccine schedules. The safety surveillance data and the results from causality assessment were shared across national decision-making bodies.
>
> We did not translate the abstract into an indigenous or local language because English is one of the languages spoken in Uganda. However, we opted to translate the abstract into French because the results of this study are relevant for many Francophone countries who have or will introduce nOPV2.

7. How were individuals, communities, and environments protected from harm?

a) *How did you ensure that sensitive patient data was handled safely and respectfully? Was there any potential for stigma or discrimination against participants arising from any of the procedures or outcomes of the study?*

Participant level data collected in the CEM was done using either ODK Collect which encrypts the data using the standard ODK encryption protocols or on paper which were kept in a locked cabinet in a secure room in-country. All digitized data were kept on password-protected computers. Data were kept confidential with no identifying personal information available to CDC staff.

b) *Might any of the tests be experienced as invasive or culturally insensitive?*

To our knowledge, no tests or questions were deemed as invasive or culturally insensitive.

c) *How did you determine that work was sensitive to traditions, restrictions, and considerations of all cultural and religious groups in the study population?*

Community leaders were socialized to the proposed activities ahead of enumerators enrolling selected households into the CEM study.

d) *Were biowaste and radioactive waste disposed of in accordance with local laws?*

Not applicable to CEM. All stool specimens were handled through routine AFP surveillance and were disposed of according to Uganda Virus Research Institute standard guidelines.

e) *Were any structures built that would have impacted members of the community or the environment (such as handwashing facilities in a public space)? If so, how did you ensure that you had appropriate community buy-in?*

Not applicable.

f) *How might the study have impacted existing health-care resources (such as staff workloads, use of equipment that is typically employed elsewhere, or reallocation of public funds)?*

This work was largely carried out during an outbreak response SIA and during the 6 weeks following the SIA. There may be disruptions to routine immunization services as healthcare workers and resources are used to support outbreak response activities. At the same time, there may be a positive impact because a house-to-house vaccination campaign may increase awareness of immunization or other health services.<br><br>Global guidelines for AFP outline that all AFP cases should be investigated within 48 hours of notification so this would be standard practice, but there was an increase in workload to the

district surveillance officer to also complete the adverse events of special interest case investigation form.

This study provided financial support for the causality assessments but the National AEFI Committee is made up of experts from across the medical and public health domains and there was considerable human resource efforts dedicated to completing the causality assessments.

8. Finally, please provide the title (eg, Dr/Prof, Mr/Mrs/Ms/Mx), name, and email address of an author who can be contacted about this statement. This can be the corresponding author.

Name: Ashley T. Longley
Email: ALongley@cdc.gov
