# Supplementary material for: Safety of nOPV2 administered during a supplementary immunisation activity in Uganda, 2022: data triangulation from a prospective cohort event monitoring programme and vaccine safety surveillance reports
Source: Lancet Glob Health. Author manuscript; Available in PMC 2025 Jul 2. (PMC12213129; doi:10.1016/S2214-109X(25)00110-X)
Supplement: SM2 [file NIHMS2090134-supplement-SM2.pdf]

THE LANCET
Global Health

Supplementary appendix 1

This translation in French was submitted by the authors and we reproduce it as supplied. It has not been peer reviewed. *The Lancet*'s editorial processes have only been applied to the original in English, which should serve as reference for this manuscript.

Cette traduction en français a été proposée par les auteurs et nous l'avons reproduite telle quelle. Elle n'a pas été examinée par des pairs. Les processus éditoriaux du *Lancet* n'ont été appliqués qu'à l'original en anglais et c'est cette version qui doit servir de référence pour ce manuscrit.

Supplement to: Longley AT, Nsubuga F, Gilani Z, et al. Safety of nOPV2 administered during a supplementary immunisation activity in Uganda, 2022: data triangulation from a prospective cohort event monitoring programme and vaccine safety surveillance reports. *Lancet Glob Health* 2025; published online May 22. https://doi.org/10.1016/S2214-109X(25)00110-X.

Innocuité du nouveau vaccin antipoliomyélitique oral de type 2 (nVPO2) administré lors des activités de vaccination supplémentaire en Ouganda, 2022 : triangulation des données d'une étude prospective de suivi des événements de cohorte et des rapports de surveillance sur la sécurité vaccinale

Résumé

Contexte

En novembre 2020, l'Organisation mondiale de la Santé (OMS) a autorisé l'utilisation du nouveau vaccin antipoliomyélitique oral de type 2 (nVPO2) dans le cadre de l'autorisation d'utilisation d'urgence en réponse à des flambées de poliovirus circulant de type 2 dérivé de souche vaccinale (PVDVc2). Bien qu'aucune préoccupation n'ait été identifiée lors des essais cliniques du nVPO2, le Comité consultatif mondial de l'OMS pour la sécurité des vaccins a requis des données plus complètes sur la sécurité du vaccin en cas d'utilisation d'urgence. Le ministère ougandais de la Santé a déclaré une épidémie de PVDVc2 en 2021 et a réagi par une campagne nVPO2 en janvier, 2022. Plus de neuf millions d'enfants âgés de 0 à 59 mois ont été vaccinés, ce qui a permis d'obtenir des données fiables sur l'innocuité du vaccin.

Méthodes

Nous avons procédé au suivi la sécurité du nVPO2 pendant 42 jours après la vaccination en utilisant : une surveillance passive de routine des manifestations post-vaccinales indésirables (MAPI) ; une surveillance continue de la paralysie flasque aiguë (PFA) ; la surveillance active en milieu hospitalier des événements indésirables d'intérêt particulier (EIIP) ; et une surveillance active des événements de cohorte (CEM). Les cas de PFA ont été examinés par le Comité national d'experts de la poliomyélite, et les cas graves de MAPI, d'EIIP et de PFA ont fait l'objet d'une évaluation de causalité par le Comité national MAPI.

Résultats

Dans l'ensemble des systèmes de surveillance, 1 128 enfants vaccinés avec le nVPO2 ont présenté une ou plusieurs MAPI : 43 enfants identifiés par la surveillance passive, 128 cas suspects de PFA, 5 cas d'EIIP, et 952 enfants ayant signalé un MAPI par le biais de la CEM. Dans l'ensemble, 109 événements indésirables ont été considérés comme graves ; 6 (fièvre, gastro-entérite (n=3), encéphalomyélite aiguë disséminée et encéphalite) ont été jugés par le Comité comme compatibles avec une association causale avec la vaccination par le nVPO2. Aucun cas de poliomyélite paralytique associée au vaccin n'a été détecté. Un décès a été détecté, jugé non cohérent avec une association causale à la vaccination avec le nOPV2, selon le Comité national MAPI.

Interprétation

Aucun nouveau problème de sécurité n'a été identifié avec l'utilisation du nVPO2 en Ouganda à la suite d'une campagne nationale de vaccination, ce qui a permis d'obtenir des données fiables pour la préqualification du produit par l'OMS et l'octroi d'une licence.

Financement

Centres de contrôle et de prévention des maladies
