# Supplementary material for: Safety of nOPV2 administered during a supplementary immunisation activity in Uganda, 2022: data triangulation from a prospective cohort event monitoring programme and vaccine safety surveillance reports
Source: Lancet Glob Health. Author manuscript; Available in PMC 2025 Jul 2. (PMC12213129; doi:10.1016/S2214-109X(25)00110-X)
Supplement: SM3 [file NIHMS2090134-supplement-SM3.pdf]

THE LANCET
Global Health

Supplementary appendix 2

This appendix formed part of the original submission and has been peer reviewed. We post it as supplied by the authors.

Supplement to: Longley AT, Nsubuga F, Gilani Z, et al. Safety of nOPV2 administered during a supplementary immunisation activity in Uganda, 2022: data triangulation from a prospective cohort event monitoring programme and vaccine safety surveillance reports. *Lancet Glob Health* 2025; published online May 22. https://doi.org/10.1016/S2214-109X(25)00110-X.

Supplementary Table 1. Comparison of demographic information, immunisation factors, and clinical characteristics among AFP cases with and without nOPV2 receipt, nOPV2 supplemental immunisation activity—Uganda, January, 2022

	**nOPV2 receipt N=126**	**No or unknown nOPV2 receipt N=33**
**Demographics**	n (%) or median (IQR)	n (%) or median (IQR)
Median age (months)	30 (18-42)	25 (15-38)
Age group (months)		
0–11	17 (13)	6 (18)
12–23	21 (17)	8 (24)
24–59	88 (70)	19 (58)
Male sex	76 (60)	15 (45)
**Routine immunisation (RI)**		
Median OPV doses in RI	3 (3-3)	0 (2-3)
Number of children without documented OPV in RI	17 (14)	8 (24)
**Clinical characteristics**[*]		
Median time between nOPV2 receipt and paralysis onset	21 (8-31)	27 (9-44)
Fever at onset	122 (97)	32 (97)
Progression to paralysis within 3 days of symptom onset[†]	122 (97)	32 (97)
Asymmetrical paralysis[‡]	102 (81)	26 (79)
Non-polio enterovirus isolated from stool	8 (6)	4 (12)

AFP=acute flaccid paralysis. IQR=interquartile range. nOPV2=novel oral poliovirus vaccine type 2. RI=routine immunisation. OPV=oral poliovirus vaccines.

[*] Less than 1% of all polio infections in children result in flaccid paralysis

[†] The clinical course may be biphasic in children, with initial minor illness that lasts several days, a symptom-free period of 1 to 3 days, followed by the major illness with paralysis, fever and muscle pain

[‡] Paralysis caused by poliovirus is typically asymmetrical

Supplementary Table 2. Characteristics of children hospitalized for symptoms of reported AEFI through cohort event monitoring, n=22, nOPV2 supplemental immunisation activity—Uganda, January, 2022

**Characteristic**	**n (%) or median (IQR)**
Age group (months)	26 (12-43)
0–11	5 (23)
12–23	6 (27)
24–59	11 (50)
Male sex	13 (59)
Pre-existing medical condition	6 (27)
Previous allergic reaction to a vaccine	5 (23)
Outcome	
Died	1 (5)
Hospitalized*	21 (95)
Hospitalized within 7 days of nOPV2 vaccination	15 (68)
Most reported final diagnosis	
Malaria	5 (23)
Acute febrile illness	3 (14)
Pneumonia	3 (14)

AEFI=adverse events following immunisation. IQR=interquartile range. nOPV2=novel oral poliovirus vaccine type 2.

* One participant died before hospital admission

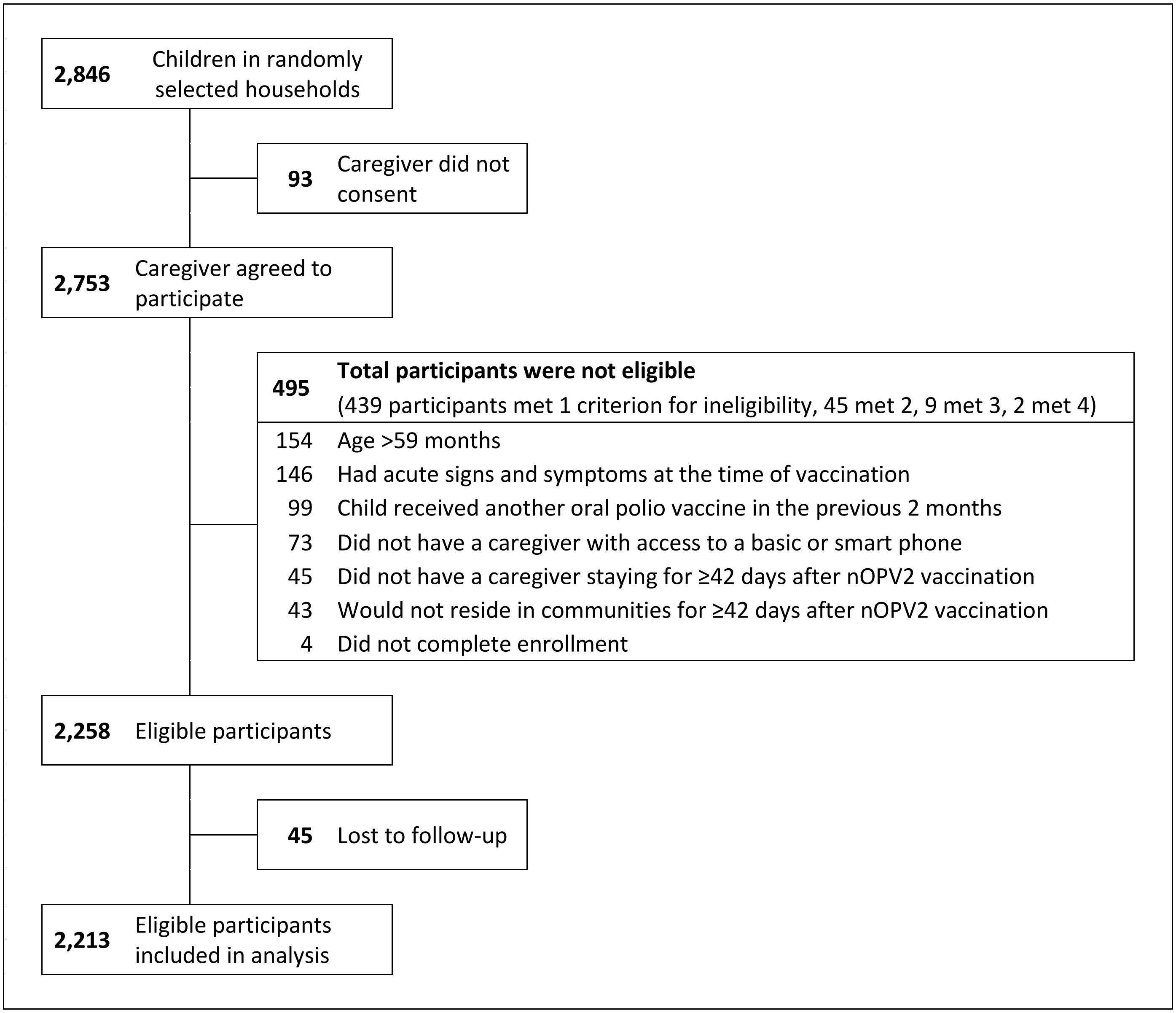

Supplementary Figure. Cohort event monitoring enrollment flow diagram, nOPV2 supplemental immunisation activity—Uganda, January, 2022
nOPV2=novel oral poliovirus vaccine type 2
